# Physiological Characterization and Comparative Transcriptome Analysis of a Slow-Growing Reduced-Thylakoid Mutant of Chinese Cabbage (*Brassica campestris* ssp. *pekinensis*)

**DOI:** 10.3389/fpls.2016.00003

**Published:** 2016-01-26

**Authors:** Shengnan Huang, Zhiyong Liu, Danyang Li, Runpeng Yao, Li Hou, Xiang Li, Hui Feng

**Affiliations:** Department of Horticulture, Shenyang Agricultural UniversityShenyang, China

**Keywords:** Chinese cabbage, slow-growing mutant, physiological characterization, transcriptome analysis, DEGs

## Abstract

Mutants are ideal for studying physiological processes. The leaves of Chinese cabbage are a major place for photosynthesis, and the mutation of these leaves may directly affect the rate of plant growth and development, thus influencing the formation of its leafy head. We characterized a slow-growing mutant, which was designated as *drm*. The *drm* exhibited slow growth and development at the seedling and heading stages, leading to the production of a tiny, leafy head, and chlorophyll-deficient leaves, especially in seedlings. Genetic analysis indicated that the phenotype of *drm* was controlled by a single recessive nuclear gene. Compared with the wild-type “FT” line, the *drm*'s chlorophyll content was significantly reduced and its chloroplast structure was abnormal. Moreover, its photosynthetic efficiency and chlorophyll fluorescence parameters were significantly decreased. The changes in leaf color, combined with these altered physiological characters, may influence the growth and development of plant, ultimately resulting in the slow-growing phenotype. To further understand the molecular regulation mechanisms of phenotypic differences between “FT” and *drm*, comparative transcriptome analyses were performed using RNA-Seq; a total of 338 differentially expressed genes (DEGs) were detected between “FT” and *drm*. According to GO and KEGG pathway analysis, a number of DEGs involved in chlorophyll degradation and photosynthesis were identified, such as chlorophyllase and ribulose-1,5-bisphosphate carboxylase/oxygenase. In addition, the expression patterns of 12 DEGs, including three chlorophyll degradation- and photosynthesis-related genes and nine randomly-selected genes, were confirmed by qRT-PCR. Numerous single nucleotide polymorphisms were also identified, providing a valuable resource for research and molecular marker-assistant breeding in Chinese cabbage. These results contribute to our understanding of the molecular regulation mechanisms underlying growth and development and lay the foundation for future genetic and functional genomics in Chinese cabbage.

## Introduction

Plant growth and development is an extremely complicated physiological and biochemical process that includes the phases of seed germination, young panicle formation, flowering, pollination, fertilization, fruiting, and aging. Each phase involves different metabolic changes and is affected by internal and external factors, primarily including the cell cycle (den Boer and Murray, 2000), light (light intensity and light quality) (Fukuda et al., 2002), and plant hormones (Lam, 2004). Plant hormones are crucial for plant growth and development, as they are involved processes throughout the plant's lifecycle and they regulate all aspects of plant growth and development; such phytohormones include IAA (Auxin; Fukuda, 2004), CTK (cytokinin; Lejeune et al., 1994), GA (gibberellin; Yamaguchi, 2008), ABA (abscisic acid; Cheng et al., 2002), and JA (jasmonic acid; Satora and Kayoka, 2001). Numerous genes related to plant growth and development have recently been discovered. For example, in *Arabidopsis thaliana, URO*, which is involved in the IAA and ethylene pathways, regulates plant growth and development (Guo et al., 2004). In maize, overexpression of *ABP9* increases plant resistance to adverse conditions and inhibits plant growth and development under normal growth conditions (Meng et al., 2007). In addition, numerous senescence-associated genes (Sags) have also been identified, such as *petB, psbA, SAG12* (Lohman et al., 1994), and *LSC54* (Buchanan-Wollaston, 1994).

The leaves are the major organs of photosynthesis, and mutations of leaves may significantly influence the photosynthetic efficiency and even the growth and development of a plant. Leaf-color mutants are often produced during physical and chemical mutagenesis and the process of tissue culture. Normal leaf color is the result of long-term evolution in plants. However, leaf-color mutations can directly or indirectly affect chlorophyll biosynthesis and degradation, resulting in reduced photosynthetic efficiency, slow growth, and development and declining competitiveness, and they can even cause plant death (Jung et al., 2003; Ihnatowicz et al., 2004; Sugimoto et al., 2004; Chen et al., 2005; Nagata et al., 2005). Leaf-color mutants are also known as Chl-deficient mutants (Liu et al., 2012). Chlorophyll-deficient mutants are widely used to study the molecular regulation mechanisms of chlorophyll biosynthesis and chloroplast development in plants (Carol et al., 1999; Isaacson et al., 2002; Lonosky et al., 2004; Noutoshi et al., 2005; Rzeznicka et al., 2005). It has been reported that a chlorophyll-deficient mutant, which was obtained by treatment with the chemical mutagen ethyl methanesulfonate (EMS), facilitated the understanding of the biological processes of chloroplast development in *Brassica napus* (Zhu et al., 2014). Therefore, leaf color is not only a useful marker character for the utilization of heterosis, but it is also an important trait for studies of photosynthesis, chlorophyll biosynthesis, chloroplast genetic transformation and development, and gene expression and regulation.

The transcriptome comprises the complete set of transcripts from a certain type of cell or tissue at a specific developmental stage or physiological condition. Transcriptome analysis can provide a comprehensive understanding of molecular mechanisms involved in specific biological processes and diseases based on gene function and structure. Next generation sequencing (NGS) technology, i.e., RNA-Seq, has developed rapidly and has been extensively applied to transcriptomics studies. This high-throughput, low cost technique is widely used to discover new genes and transcripts in plants (Wall et al., 2009; Huang and Khatib, 2010; Garg et al., 2011), to develop Single Nucleotide Polymorphisms (SNPs) and molecular markers (Novaes et al., 2008; Cánovas et al., 2010; Montgomery et al., 2010; Hansey et al., 2012), to analyze gene expression profiles (Bruno et al., 2010; Severin et al., 2010), to identify differentially expressed genes (DEGs; Camarena et al., 2010; Faulconnier et al., 2011) and in RNA editing (Peng et al., 2012), alternative splicing (AS; Filichkin et al., 2010; Zenoni et al., 2010; Wang et al., 2011a), and metabolic pathway analysis (Barrero et al., 2011; Feng et al., 2012). These studies have provided massive amounts of sequencing data for studying gene expression patterns and for DEG identification and molecular marker development.

Chinese cabbage (*Brassica campestris* ssp. *pekinensis* [Lour] Olsson), which belongs to the family Brassicaceae, is an economically important, popular vegetable crop that is widely cultivated in Asia. The size of leafy head is an important indicator for measuring the yield and quality of Chinese cabbage. Changes in leaf color may affect the rate of plant growth and development and even the formation of its leafy head, ultimately influencing yields. The completion of genome sequencing of Chinese cabbage (Wang et al., 2011b) has paved the way for comparative transcriptome analysis and functional genomics studies of this crop.

In this study, we characterized a slow-growing mutant, which was designated as *drm*. The *drm* exhibited slow growth and development and chlorophyll-deficient leaves. Comparative physiological studies were conducted to investigate the phenotypic differences between *drm* and the wild-type “FT” line. Moreover, to obtain a comprehensive and integrated genomic resource and to better elucidate the gene expression patterns in *drm*, we utilized Illumina NGS technology to compare the transcriptome profiles of *drm* and “FT.” Numerous DEGs were identified, including a number of genes related to chlorophyll degradation and photosynthesis. The results provide a comprehensive view of the transcriptome of *drm*, which contributes to our understanding of the important metabolic or regulatory mechanisms underlying the growth and development of Chinese cabbage. The results also represent a valuable resource for future genetic and genomic studies in Chinese cabbage.

## Materials and methods

### Plant materials

The *drm* was screened by Huang et al. (2014) and derived from Chinese cabbage DH line “FT” by a combination of isolated microspore culture and radiation treatment (^60^Co γ-rays). This mutant was diploid as determined by flow cytometry and exhibited stable inheritance after multiple generations. It is assumed that the genetic background between “FT” and *drm* is completely consistent, with the only difference found at the mutation sites.

### Genetic analysis

To study the genetic patterns of *drm*, a reciprocal cross between *drm* and the wild-type “FT” line was performed. The BC_1_ generation was obtained by backcrossing of F_1_ to *drm* and “FT.” Also, the F_2_ population was derived from self-pollination of F_1_ plants. The phenotype of the reciprocal hybrid F_1_ and the segregation ratios of the BC_1_ and F_2_ populations were recorded. The data from these experiments were analyzed using the Chi-square (χ^2^)-test.

### Investigation of agronomic traits

In August 2014, “FT” and *drm* seeds were sown in a greenhouse at Shenyang Agricultural University. At the seedling stage, when the third true leaves appeared, the leaf length, leaf width, leaf index of the third true leaves, and plant width were measured in for both lines every 3 days until leafy heads began to form; the plants were measured 12 times. At the heading stage, when the leafy head of each plant was mature, the head weight, head length, head width, and head length/head width ratio of “FT” and *drm* were measured. Five consistent and robust “FT” and *drm* plants were, respectively, selected at the seedling and heading stage; each measurement was performed in three independent experiments.

### Measurement of chlorophyll and carotenoid contents

Consistent, robust “FT” and *drm* plants at the seedling stage were also selected for chlorophyll and carotenoid measurements. When the third true leaves appeared, chlorophyll and carotenoid in the third true leaves of “FT” and *drm* were extracted with 90% (v/v) acetone solution every 3 days according to the method of Arnon (1949). The absorbance was recorded at 663, 645, and 470 nm using a UV spectrophotometer (T6 New Century, Persee, Beijing, China); each measurement was repeated three times. The total chlorophyll, chlorophyll a, chlorophyll b and carotenoid contents were calculated based on these measurements.

### Measurement of photosynthetic characters

Ten consistent, robust “FT” and *drm* plants were selected at the seedling stage, respectively. The net photosynthetic rate (A), stomatal conductance (G_*H*2*O*_), intercellular CO_2_ concentration (Ci), and transpiration rate (E) of the fully-expanded third true leaves were measured in the field using the GFS-3000 portable photosynthesis system (WALZ, Germany) from 9:00 to 11:00 a.m. The temperature was set at 30°C, CO_2_ concentration was subjected to external conditions, and simulation light intensity was set at 1200 μmol·m^−2^·S^−1^. Each plant was measured three times.

### Measurement of fluorescence kinetic parameters

A Fluor Cam Portable ChI/GFP Luminoscope (Handy GFPCam; Eco Tech, Beijing, China) was used to measure the chlorophyll fluorescence kinetic parameters of the fully-expanded third true leaves of “FT” and *drm* at the seedling stage according to the method of Feng et al. (2008). Five consistent, robust plants were, respectively, selected from “FT” and *drm*; each measurement was repeated three times. The dark treatment was initially conducted for 30 min. Minimal fluorescence (*F*_0_), optional maximal photochemical efficiency of PS II (Fv/Fm), actual photochemical efficiency of PSII (Φ_PSII_), photochemical quenching (qP), and non-photochemical quenching (NPQ) were subsequently measured.

### Observing chloroplasts using transmission electron microscopy

Fresh third true leaves (avoiding large veins) of “FT” and *drm* at the same position were selected from seedlings growing in the field. For transmission electron microscopy analysis, the leaves were cut into 1 mm^2^ sections, fixed in glutaraldehyde solution (2.5% glutaraldehyde, 0.1 mol·L^−1^ Na_2_HPO_4_, 0.1 mol·L^−1^ NaH_2_PO_4_, pH 7. 0), washed in 0.1 mol·L^−1^ phosphate buffer, further fixed in 1% OsO_4_ in the same phosphate buffer, and dehydrated step-by-step with various concentrations of acetone, followed by embedding and polymerization using Epon812. After ultrathin sectioning (LKB2088 type ultramicrotome, LKB Company, Sweden), the samples were double-dyed with uranyl acetate and lead citrate solutions and observed under a transmission electron microscope (H-7650; Hitachi, Japan); photographs were taken at 30,000-fold magnification (Hao et al., 2010; Ning et al., 2012).

### RNA extraction

To further understand the molecular regulation mechanisms of the phenotypic differences, comparative transcriptome analyses were performed between “FT” and *drm*. In October 2014, “FT” and *drm* seeds were sown in a greenhouse at Shenyang Agricultural University. The third true leaves of “FT” and *drm* were collected as described above and used as transcriptome sequencing materials during the optimal period in which the most significant differences in growth and development were observed. All samples were immediately frozen in liquid nitrogen and stored at –80°C until further processing. Mixed samples collected from five “FT” or *drm* plants were used as one biological replicate; three independent biological replicates were prepared for “FT” and *drm*.

Total RNA from these six samples was extracted using Trizol Reagent (Invitrogen, USA) according to the manufacturer's instructions. RNA quality and purity were assessed using an Agilent 2100 Bioanalyzer (Agilent, USA), and the integrity of all RNA samples was monitored by 1.0% agarose gel electrophoresis.

### Construction of cDNA libraries and illumina sequencing

To construct six cDNA libraries, equal amounts of total RNA from the three independent biological replicates of “FT” and *drm* were pooled for RNA-Seq library construction, which were designated as FT2, FT4, FT5, DRM2, DRM4, and DRM5, respectively. Poly (A) mRNA was purified from total RNA using oligo (dT) magnetic beads and fragmented into small pieces by the addition of fragmentation buffer. These short fragments served as templates to synthesize first-strand cDNA using random hexamer-primers. Second-strand cDNA was synthesized using buffer, dNTPs, RNaseH and DNA polymerase I. Short fragments were purified using a QiaQuick PCR extraction kit. These fragments were washed with EB buffer for end-repair/poly (A) addition and ligated to sequencing adapters. Suitable fragments (as judged by agarose gel electrophoresis) were selected for use as templates for PCR amplification. The Agilent 2100 Bioanalyzer and ABI StepOnePlus Real-Time PCR System were employed for quantification and qualification of the constructed library. The library products were then subjected to sequencing analysis via Illumina HiSeq™ 2000 platform.

### Mapping reads to the reference genome

The original image data were converted into sequence data by base calling. Low-quality reads were omitted from data analysis. Low-quality reads were defined as (i) reads in which more than 50% of bases had a quality value ≤ 10, (ii) reads in which more than 10% of bases were unknown and (iii) reads with adaptors. Clean reads filtered from raw reads were mapped to the reference genome using SOAPaligner/SOAP2 (Li et al., 2009). Mismatches of no more than five bases were allowed in the alignment.

In order to further characterize gene structures, the 5′- and 3′-gene boundaries were more precisely defined by investigating the upstream and downstream regions of the existing genes, which were predicted in the genome (Roberts et al., 2011).

### Identification and functional enrichment analysis of DEGs

Gene expression levels were calculated using the RPKM (Reads Per kb per Million reads) method (Mortazavi et al., 2008), which eliminated the influence of different gene lengths and sequencing discrepancies on the calculation of gene expression level. DESeq was applied to analyze DEGs based on the RPKM-derived baseMean for each gene between samples (Anders and Huber, 2010). To identify DEGs, the False Discovery Rate (FDR) method was used to determine the threshold of *P*-value in multiple tests (Benjamini and Hochberg, 1995). In this study, genes with the absolute value of log_2_ Ratio ≥ 5 and FDR ≤ 0.001 were defined as DEGs. In addition, SEGs were defined as those were not expressed in one library but whose baseMean values were greater than 11 in the other library (Tao et al., 2012).

Gene Ontology (GO) analysis (Ashburner et al., 2000), an international standardized gene functional classification system, offers a dynamically updated controlled vocabulary and a strictly defined way to comprehensively describe the properties of genes and their products. To perform complete GO function analysis, the DEGs were characterized into three categories: biological process, cellular component and molecular function. Pathway-based analysis helps further elucidate the biological functions of genes; Kyoto Encyclopedia of Genes and Genomes (KEGG; Kanehisa et al., 2008) is a major public pathway-related database.

The GO and KEGG pathway functional-enrichment analyses were performed to classify the DEGs. All DEGs were mapped to GO terms in the database (http://www.geneontology.org/) and pathways in the KEGG database (Kanehisa et al., 2008), respectively. Compared to the genome background, the significantly enriched GO terms and KEGG pathways in DEGs were identified using hypergeometric tests and the Bonferroni-corrected *P* ≤ 0.05 as the threshold (Abdi, 2007).

### Identification of SNPs

Since the genotype of three biological replicates of “FT” and *drm* were identical, the RNA-Seq data from the biological replicates were combined for SNPs identification. SNPs were detected using SOAPsnp software (Li et al., 2009), which was a member of the SOAP (Short Oligonucleotide Analysis Package). The SOAPsnp program was a resequencing utility, which can assemble consensus sequences for the genome/transcriptome of a newly sequenced individual based on the alignment of the sequencing reads on the known reference. Then the SNPs can be identified through the consensus sequence in comparison with the reference sequence.

### Quantitative real-time PCR (qRT-PCR) analysis

Total RNA was extracted from the same plant samples (“FT” and *drm*) as those used for RNA-Seq using Trizol Reagent (Invitrogen, USA), and first-strand cDNA was synthesized using a FastQuant First Strand cDNA Synthesis kit (Tiangen, Beijing, China) according to the manufacturer's instructions. To confirm the gene expression patterns of the DEGs, a total of 12 DEGs, including three chlorophyll degradation- and photosynthesis-related genes (*Bra025756, Bra025431*, and *Bra028406*) and nine randomly-selected genes (*Bra031329, Bra037196, Bra014819, Bra015728, Bra039414, Bra003874, Bra007953, Bra029272*, and *Bra011660*), were analyzed using qRT-PCR. The qRT-PCR was performed on a Bio-Rad IQ5 Real Time PCR System (Bio-Rad, USA) using SYBR Green PCR master mix (Takara). Each 20-μL reaction consisted of 9 μl 2.5X Real MasterMix/20X SYBR solution, 2 μL (2 μmol·L^−1^) of forward and reverse primers, 2 μl diluted cDNA (50 ng) and 5 μl ddH_2_O. The *Actin* gene was used as an internal control, and gene-specific primers were designed with Primer Premier Software (version 5.0); the primer sequences were shown in Table S7. The qRT-PCR was performed using the following reaction conditions: 95°C for 3 min, followed by 40 cycles of 95°C for 30 s, 57°C for 30 s, and 68°C for 15 s. Melting curves (55–95°C) were then performed for each reaction to detect primer dimers. The relative expression levels of genes were calculated using the 2^−Δ*ΔCt*^ method (Livak and Schmittgen, 2001). All reactions were performed with three biological replicates. The expression differences between samples were analyzed using Bio-Rad IQ5 Manager software.

## Results

### Morphological analysis of *drm* and wild-type “FT”

**Figure 1:**
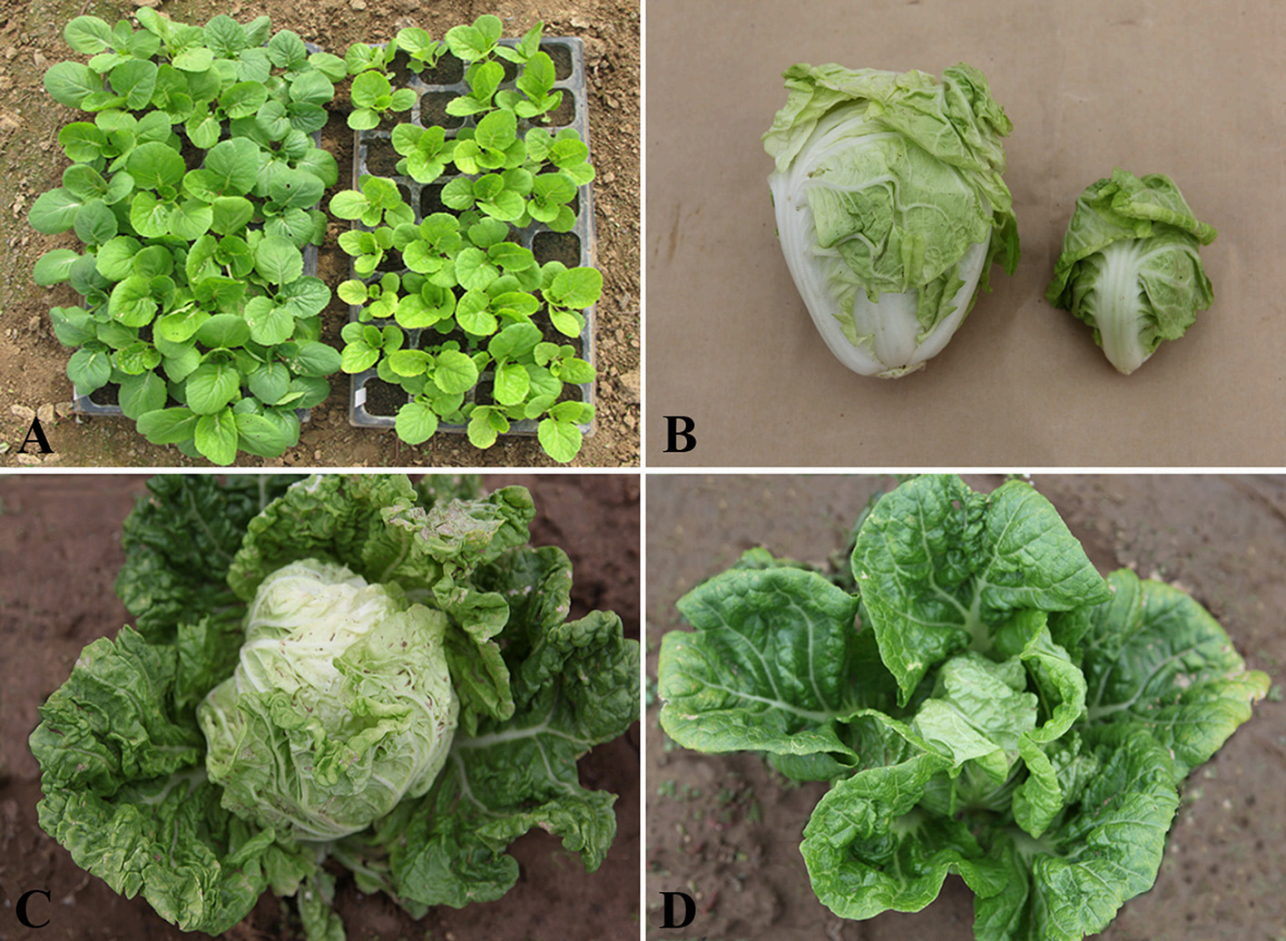
**Phenotypic characterization of *drm* and wild-type “FT.” (A)** “FT” (left) and *drm* (right) at the seedling stage; **(B)** leafy head of “FT” (left) and *drm* (right); **(C)** “FT” at the heading stage; **(D)** *drm* at the heading stage.

Compared with “FT,” *drm* exhibited slower growth and development at the seedling stage, which continued at the heading stage, eventually leading to the production of very small leafy heads. The mutant was not only slow-growing, but it also exhibited chlorophyll-deficient leaves, especially at the seedling stage (Figure 1).

### Genetic analysis of *drm*

The reciprocal cross F_1_ plants exhibited the same phenotype as “FT,” which indicates that the phenotype of *drm* is recessive and controlled by nuclear genes. The segregation ratio of F_1_ × *drm* in the BC_1_ progenies was ~1:1 [χ^2^ = 1.65 $<\chi_{\left(0.05\right)}^{2}=3.84$]. In addition, the F_2_ population showed the expected segregation ratio of 3:1 [χ^2^ = 2.58 < $\chi_{\left(0.05\right)}^{2}=3.84$]. These data indicate that the phenotype of *drm* is controlled by a single recessive gene (Table 1).

**Table 1:** **Genetic analysis of *drm* mutant and crosses between *drm* and “FT”**.

**Generation**	**Total**	**“FT”**	***drm***	**Segregation ratio**	**χ^2^**
P_1_ (“FT”)	180	180	0		
P_2_ (*drm*)	156	0	156		
F_1_ (P_1_ × P_2_)	204	204	0		
F_1_ (P_2_ × P_1_)	216	216	0		
BC_1_ (F_1_ × “FT”)	147	147	0		
BC_1_ (F_1_ ×*drm*)	144	79	65	1.22:1	1.65
F_2_	207	165	42	3.93:1	2.58

### Agronomic traits analysis of *drm* and wild-type “FT”

**Figure 2:**
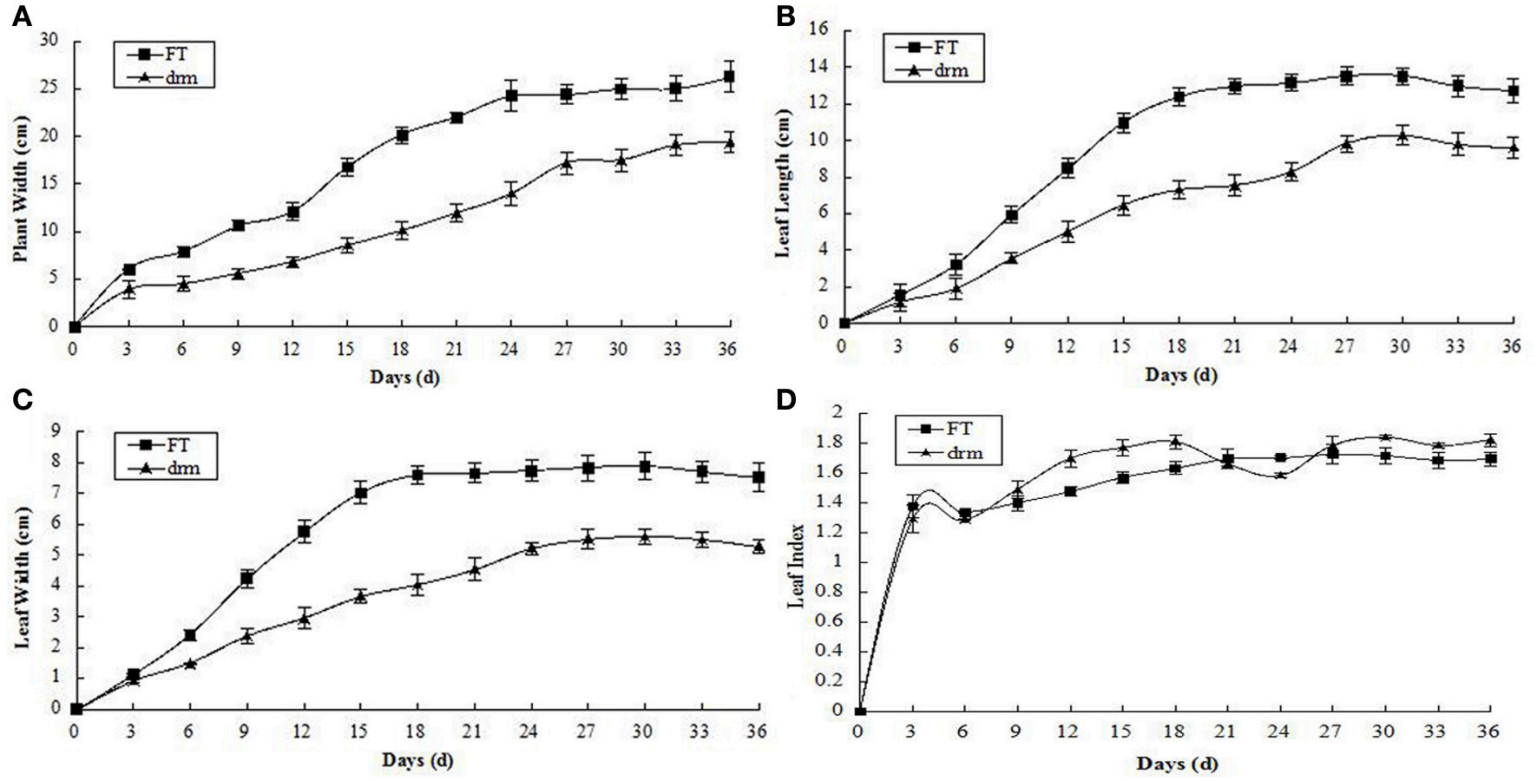
**Agronomic traits of *drm* and wild-type “FT” at the seedling stage**. **(A)** Plant width; **(B)** leaf length; **(C)** leaf width; **(D)** leaf index. Days (d): days after the third true leaves appeared. Each value is the mean of three independent experiments. The *error bars* represent standard error (SE) of the means.

At the seedling stage, the leaf length, leaf width of the third true leaves, and plant width were significantly reduced in *drm* compared to “FT,” while there was no obvious difference in leaf index between lines. In addition, 15–24 days after the third true leaves appeared, the changes in leaf length, leaf width, and plant width of both “FT” and *drm* were the most rapid, and the greatest significant quantitative differences between these values were observed between lines (Figure 2).

At the heading stage, when the leafy head was mature, the head weight, head length, and head width were obviously higher in “FT” than in *drm*, while the head length/head width ratios were similar between lines. Among these, the difference in head weight was the most obvious. The head weight of “FT” was ~3.5-times that of *drm*; this difference was highly significant (Table 2).

**Table 2:** **Agronomic and photosynthetic characters of *drm* and “FT”**.

**Traits**	**Characteristics**	**“FT”**	***drm***
Agronomic characters at the heading stage	Mean of head weight (Kg)	0.48 ± 0.05^*^	0.14 ± 0.01
	Mean of head length (cm)	14.65 ± 0.42^*^	10.70 ± 0.41
	Mean of head width (cm)	11.50 ± 0.66^*^	7.78 ± 0.49
	Mean of head length/head width ratio	1.28 ± 0.04	1.38 ± 0.03
Photosynthetic characters at the seedling stage	Net photosynthetic rate (A, μmol CO_2_m^−2^s^−1^)	17.14 ± 0.53^*^	13.68 ± 0.53
	Stomatal conductance (G_H2O_, mol H_2_O m^−2^s^−1^)	249.90 ± 21.75^*^	146.85 ± 11.99
	Intercellular CO_2_ concentration (Ci, μmol CO_2_ mol^−1^)	364.71 ± 9.88^*^	315.80 ± 6.96
	Transpiration rate (E, mol H_2_O m^−2^s^−1^)	3.34 ± 0.26^*^	2.22 ± 0.17
Chlorophyll fluorescence kinetic parameters at the seedling stage	Minimum fluorescence (*F*_0_)	127.48 ± 1.23^*^	101.85 ± 1.94
	Primary photochemical efficiency of PS II (Fv/Fm)	0.81 ± 0.02	0.78 ± 0.03
	Actual photochemical efficiency of PSII (Φ_PSII_)	0.72 ± 0.02	0.71 ± 0.03
	Photochemical quenching (qP)	0.95 ± 0.02	0.90 ± 0.03
	Non-photochemical quenching (NPQ)	0.47 ± 0.05	0.45 ± 0.02

*Means and standard error (SE)-values were calculated from three independent replicates. Data were analyzed using SPSS 16.0 (Chicago, IL, USA)*.

* *Significantly different at a level of 0.05 by t-test*.

### Chlorophyll and carotenoid content analysis of *drm* and wild-type “FT”

**Figure 3:**
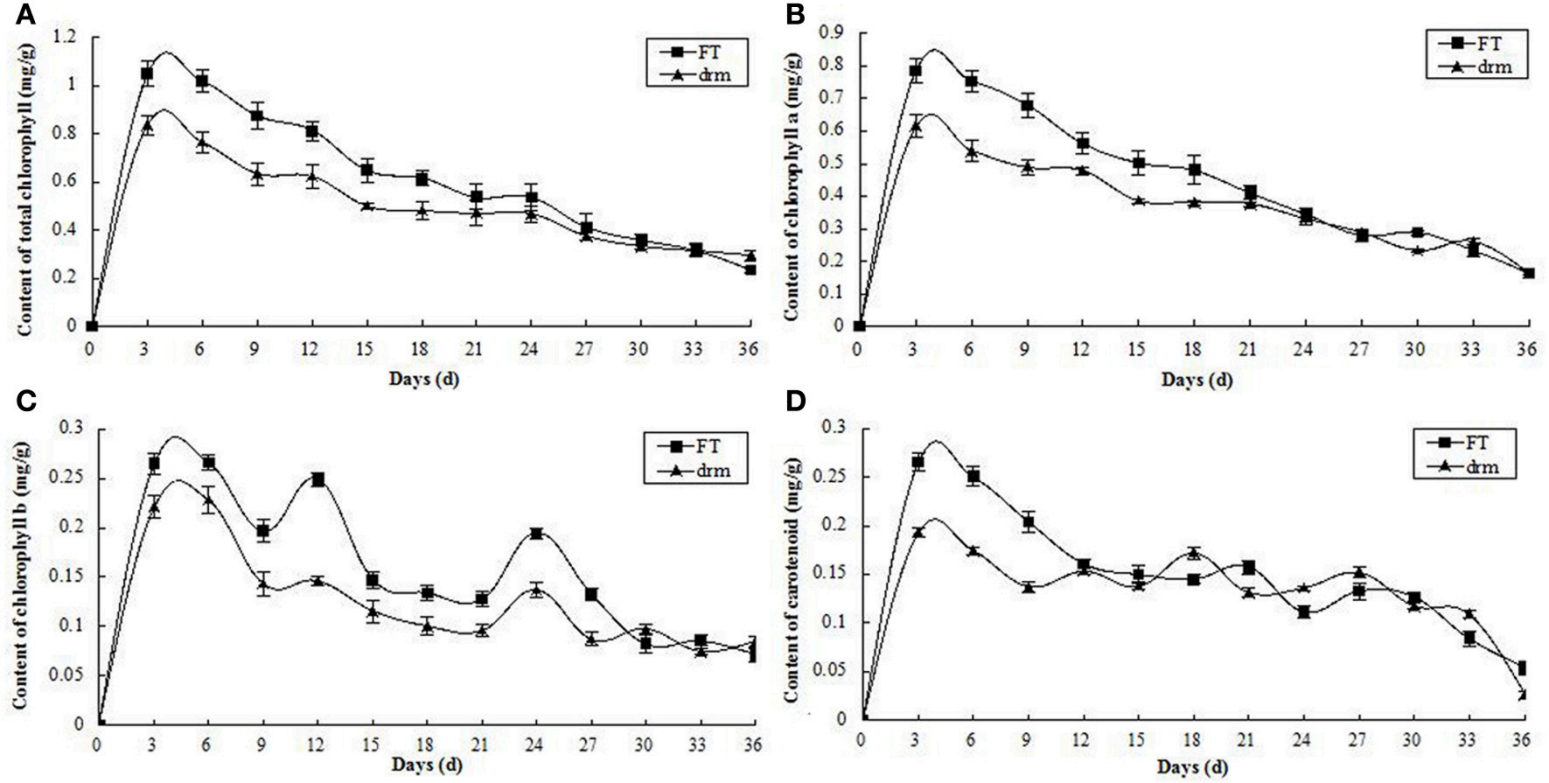
**Dynamic changes in chlorophyll and carotenoid contents in *drm* and wild-type “FT” at the seedling stage**. **(A)** total chlorophyll contents; **(B)** chlorophyll a contents; **(C)** chlorophyll b contents; **(D)** carotenoid contents. Days (d): days after the third true leaves appeared. Each value is the mean of three independent experiments. The *error bars* represent standard error (SE) of the means.

As the third true leaves developed, the contents of chlorophylls (total chlorophylls, chlorophyll a and chlorophyll b) and carotenoids gradually declined. Compared to “FT,” the chlorophyll contents were significantly reduced in *drm*, whereas the carotenoid contents were similar between lines (Figure 3). These results indicate that the reduced chlorophyll contents in *drm* are related to the chlorophyll-deficient leaf phenotype of the mutant at the seedling stage.

### Photosynthetic character analysis of *drm* and wild-type “FT”

As shown in Table 2, the net photosynthetic rate (A), stomatal conductance (G_*H*2*O*_), intercellular CO_2_ concentration (Ci), and transpiration rate (E) in the fully-expanded third true leaves of *drm* were significantly reduced compared to those of “FT.” These results suggest that *drm* has impaired chlorophyll biosynthesis, which further leads to reduced photosynthetic efficiency of this mutant.

### Fluorescence kinetic parameter analysis of *drm* and wild-type “FT”

Chlorophyll fluorescence parameters are used to describe the mechanism underlying photosynthesis as well as plant photosynthetic physiology (Zhang, 1999). Compared to “FT,” the optional maximal photochemical efficiency of PS II (Fv/Fm), actual photochemical efficiency of PSII (Φ_PSII_), photochemical quenching (qP), and non-photochemical quenching (NPQ) of *drm* were reduced; however, these reductions were not significant. Only the minimum fluorescence (*F*_0_) of *drm* was significantly reduced in the mutant (Table 2); this value is related to the concentration of chlorophyll in leaves. This difference in *F*_0_-values between *drm* and “FT” is in accordance with the reduced chlorophyll contents in *drm*.

### Observation of chloroplast ultrastructure in *drm* and wild-type “FT”

**Figure 4:**
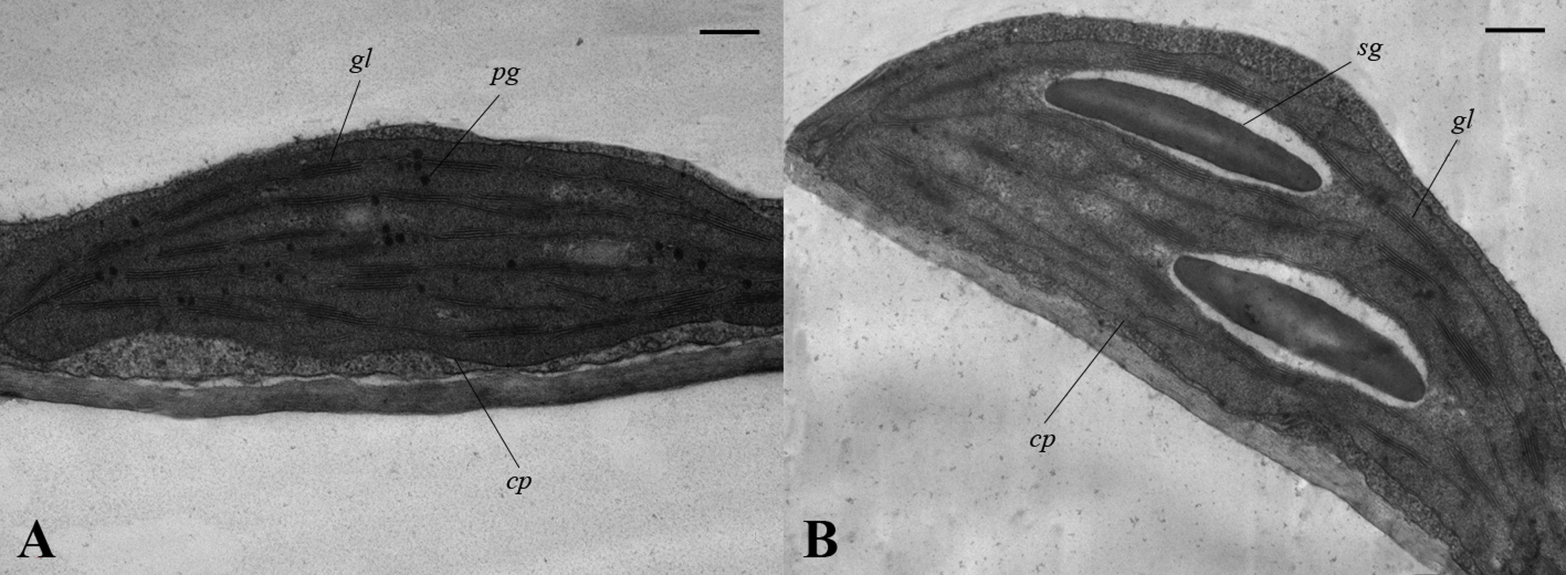
**Chloroplast ultrastructure of *drm* and wild-type “FT” at the seedling stage (× 30,000)**. **(A)** chloroplasts of “FT;” **(B)** chloroplasts of *drm*; *cp* chloroplast, *sg* starch grain, *pg* plastoglobule, *gl* grana lamella. Scale bar, 1 μm.

To investigate how chloroplast development is affected in *drm*, we compared the chloroplast ultrastructure of *drm* and “FT” using transmission electron microscopy. As shown in Figure 4, there were no apparent differences in chloroplast shape between *drm* and “FT.” “FT” chloroplasts had obvious, abundant thylakoid structures with grana and lamella; the grana and lamella stacks were well-ordered and distributed fairly evenly. In addition, a number of plastoglobules were observed. However, the chloroplasts of *drm* were less dense, with fewer thylakoid stacks of grana and lamella than those of “FT,” and the stacks were irregular and distributed unevenly, suggesting that the chloroplast structure was degraded. Also, relatively large starch grains were observed in the chloroplasts. These results indicate that *drm* does not simply exhibit reduced chlorophyll accumulation; its chloroplast development is also impaired.

### Transcriptome sequencing and comparison with the reference genome

A total of 278.9 million clean reads were generated. Of the total clean reads from the six samples, 46.1–47.2% were a perfect match, 26.2–27.3% had no more than five base mismatches, 69.1–71.4% were mapped to unique genome locations, 2.0–4.4% were mapped to multiple genome locations, and 26.2–26.7% were unmapped reads (Table 3).

**Table 3:** **Number of reads based on Illumina sequencing data in six libraries of *drm* and “FT”**.

**Reads category**	**FT2**	**FT4**	**FT5**	**DRM2**	**DRM4**	**DRM5**
Total clean reads	46,843,740	46,839,370	46,512,842	46,607,084	45,622,418	46,506,924
Total base pairs	4,215,936,600	4,215,543,300	4,186,155,780	4,194,637,560	4,106,017,620	4,185,623,160
Total mapped reads	34,362,120 (73.35%)	34,411,673 (73.47%)	34,263,571 (73.66%)	34,395,024 (73.80%)	33,447,769 (73.31%)	34,224,146 (73.59%)
Perfect match reads	21,975,803 (46.91%)	22,128,010 (47.24%)	21,880,838 (47.04%)	21,768,742 (46.71%)	21,015,055 (46.06%)	21,567,699 (46.38%)
≤ 5 bp mismatch reads	12,386,317 (26.44%)	12,283,663 (26.23%)	12,382,733 (26.62%)	12,626,282 (27.09%)	12,432,714 (27.25%)	12,656,447 (27.21%)
Unique match reads	33,420,172 (71.34%)	32,353,598 (69.07%)	32,874,184 (70.68%)	33,260,357 (71.36%)	32,263,301 (70.72%)	33,206,884 (71.40%)
Multi-position match reads	941,948 (2.01%)	2,058,075 (4.39%)	1,389,387 (2.99%)	1,134,667 (2.43%)	1,184,468 (2.60%)	1,017,262 (2.19%)
Total unmapped reads	12,481,620 (26.65%)	12,427,697 (26.53%)	12,249,271 (26.34%)	12,212,060 (26.20%)	12,174,649 (26.69%)	12,282,778 (26.41%)

*Mapped reads are the sum of perfectly matched reads and reads with ≤ 5 bp mismatch, or the sum of uniquely matched reads and multi-position matched reads. Numbers in parenthesis represent percentages of the total number of clean reads in each library*.

**Figure 5:**
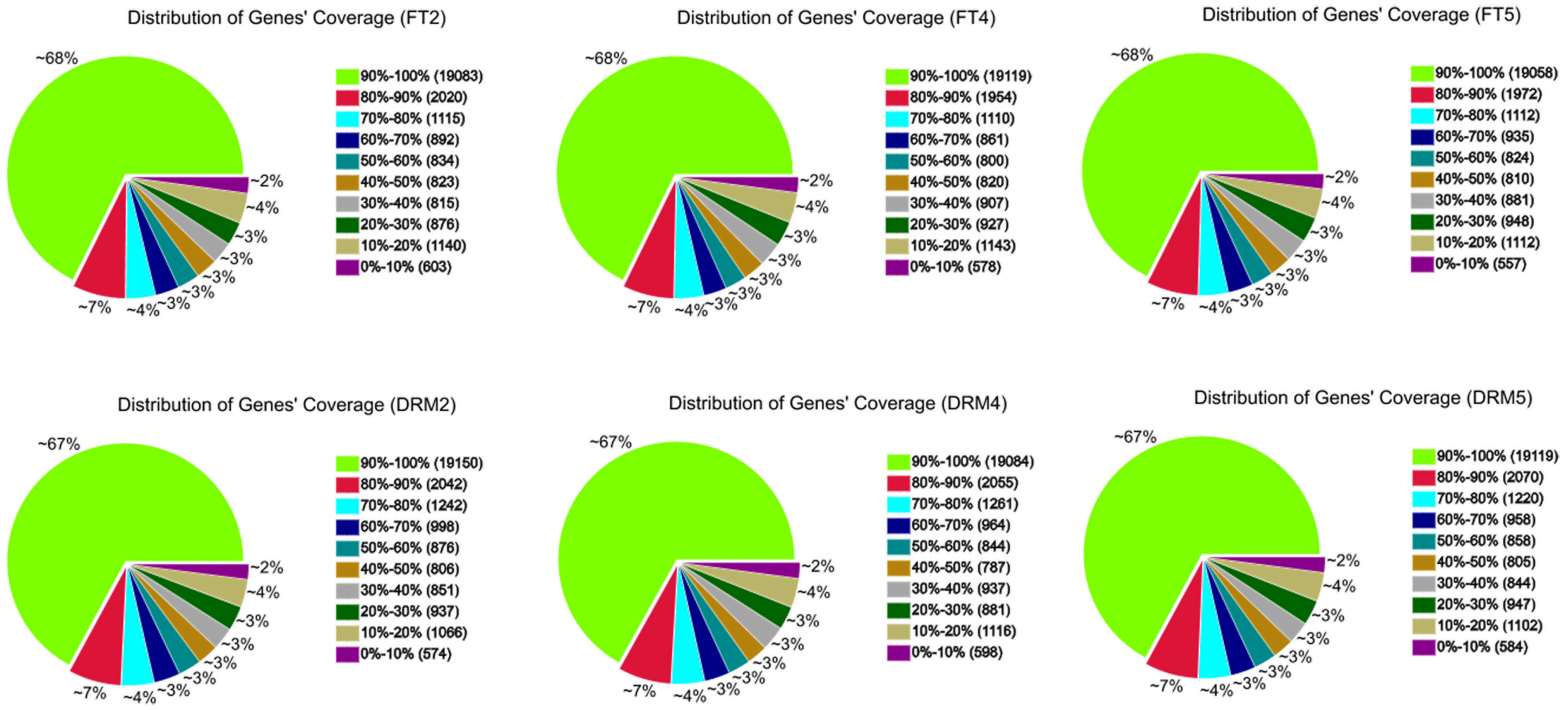
**Gene coverage of *drm* and wild-type “FT” mapped to the reference genome**. Results are shown as the percentage of the total number of genes.

All 278.9 million clean reads were mapped to 31,373 genes in the reference genome. The size distribution of all 31,373 genes detected in *drm* and “FT,” genes in the most abundant group were 500–1000 bp in length (9589; 30.6% of the 31,373 genes), followed by 1000–1500 bp (7988; 25.5% of all genes), and 100–500 bp (4764; 15.2% of all genes; Table S1). Among the six samples, there were 28,201 (FT2), 28,219 (FT4), 28,209 (FT5), 28,542 (DRM2), 28,527 (DRM4), and 28,507 (DRM5) genes, providing large amounts of data for further differential gene expression analysis. Gene coverage, i.e., the percentage of a gene covered by reads, can reflect the quality of sequencing. This value is equal to the ratio of the base number in a gene covered by unique mapping reads to the total base number in that gene (Figure 5).

In total, 3505 (FT2), 3442 (FT4), 3406 (FT5), 3516 (DRM2), 3504 (DRM4), and 3636 (DRM5) gene sequences were extended at the 5′-end, and 3511 (FT2), 3427 (FT4), 3393 (FT5), 3534 (DRM2), 3551 (DRM4), and 3675 (DRM5) gene sequences were extended at the 3′ end (Tables S2, S3).

In addition, based on the transcriptome data and annotation of reference genes, a total of 1693 and 1513 novel transcripts were detected in “FT” and *drm*, respectively. Among which, 839 (“FT”) and 794 (*drm*) novel transcripts were protein-coding, and 854 (“FT”) and 719 (*drm*) novel transcripts were non-coding (Table S4).

### Identification of SNPs

A total of 181,607 and 174,923 SNPs in “FT” and *drm* were identified, mainly including transitions and transversions (Table S5). Among which, the most common base substitution was A/G, followed by C/T, while the rarest was C/G (Table 4). In addition, the SNPs constrasting “FT” and *drm* were analyzed, of these SNPs, 7286 and 6730 were specifically detected in “FT” and *drm*, respectively.

**Table 4:** **Summary of single nucleotide polymorphism (SNP) types identified in *drm* and “FT”**.

**SNP type**	**“FT”**	***drm***
Transition	102,832 (56.62%)	99,001 (56.60%)
A/G	51,752 (28.50%)	49,863 (28.51%)
C/T	51,080 (28.13%)	49,138 (28.09%)
Transversion	78,775 (43.38%)	75,922 (43.40%)
A/C	21,003 (11.57%)	20,216 (11.56%)
A/T	20,749 (11.43%)	20,000 (11.43%)
C/G	16,832 (9.27%)	16,199 (9.26%)
G/T	20,191 (11.12%)	19,507 (11.15%)
Total	181,607	174,923

*The numbers in parentheses indicate the percentages of total number of SNPs in “FT” and drm, respectively*.

### Analysis of differential gene expression

We detected 338 DEGs between “FT” and *drm*, including 284 genes that were upregulated and 54 that were downregulated in the *drm* vs. “FT” comparison. The number of upregulated DEGs was dramatically higher than the number of downregulated DEGs (Table S6).

Numerous specifically expressed genes (SEGs) were also observed in this study. We identified 89 SEGs between “FT” and *drm*, including 24 specifically expressed in “FT” and 65 in *drm*.

Among the DEGs, we identified numerous potential stress tolerance-related genes that are responsive to drought, salt, heat, disease, or osmotic stress, such as those encoding JASMONATE-ZIM-DOMAIN PROTEIN 6 (JAZ6; *Bra016056*), dehydration-responsive protein-related (*Bra035023*), late embryogenesis abundant 3 family protein (LEA3; *Bra033350*), disease resistance family protein (*Bra026368* and *Bra039414*), F-box protein (*Bra000832*), and heat shock protein binding (*Bra007953*). Most of these genes were highly expressed, and their expression patterns differed between “FT” and *drm* (Table S6).

### DEGs related to chlorophyll degradation and photosynthesis

All of the physiological analyses suggested that the slow growth of the mutant resulted from a significant decline in physiological features, especially the reduction in chlorophyll content. The presence of the mutant gene in *drm* likely impeded chlorophyll biosynthesis, which would further influence photosynthetic efficiency, eventually leading a reduce rate of growth and development.

Among the DEGs, a number of chlorophyll degradation and photosynthesis-related genes were identified, including genes for chlorophyllase (*Bra025756*) and ribulose-1,5-bisphosphate carboxylase/oxygenase (Rubisco; *Bra028406* and *Bra025431*). Among which, Rubisco, a crucial enzyme in photosynthesis, influences the photosynthetic efficiency and thus, regulates plant growth and development (Evans, 1986); Chlorophyllase is a key enzyme in chlorophyll metabolism, especially chlorophyll degradation (Hörtensteiner, 1999). In the *drm* vs. “FT” comparison, these genes were all upregulated and had relatively high expression levels (Table S6).

### GO and KEGG pathway enrichment analysis of DEGs

**Figure 6:**
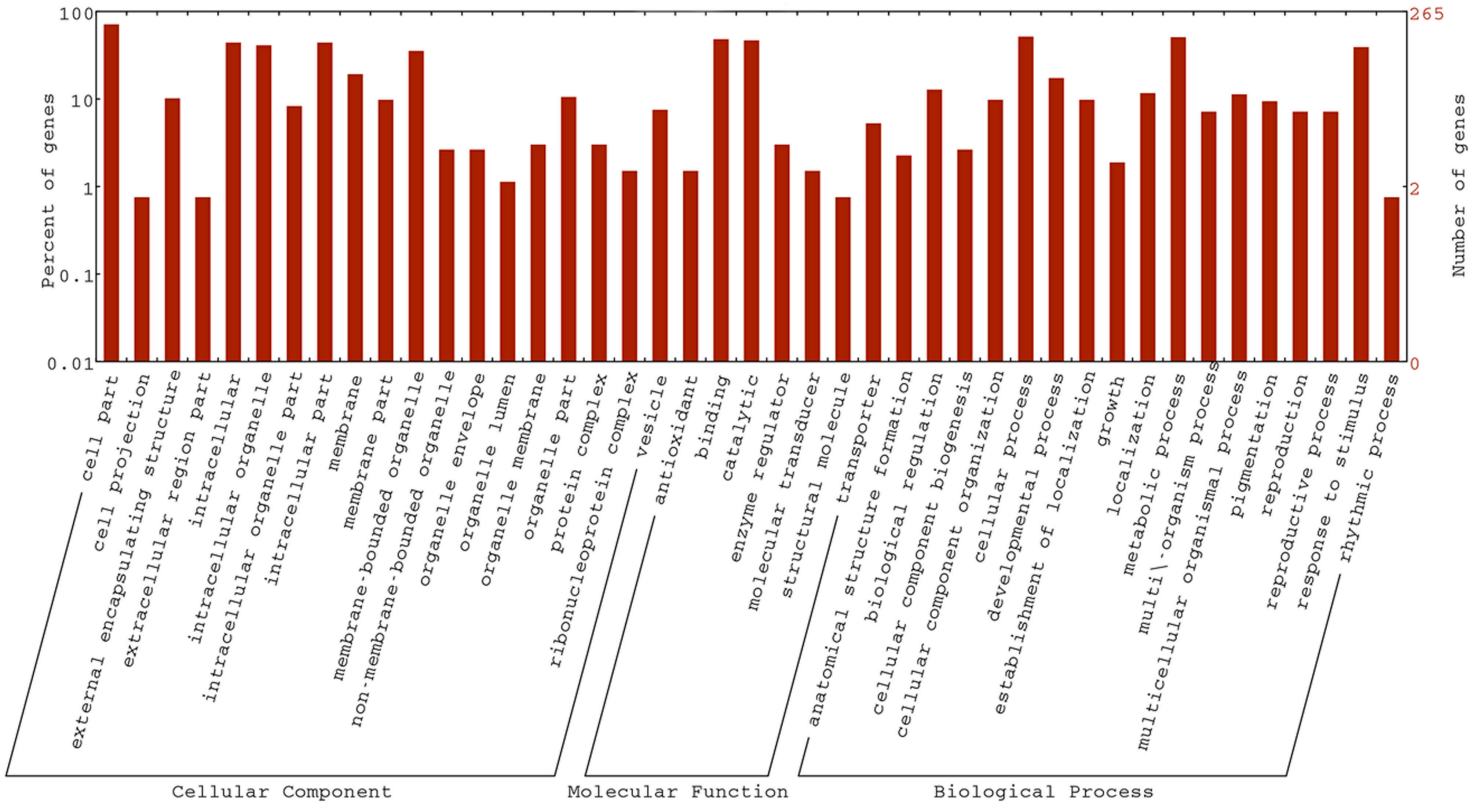
**GO classifications of DEGs in *drm* vs. “FT”**.

In the *drm* vs. “FT” comparison, a total of 266 DEGs (78.70%) were assigned to at least one GO term, including 212 (62.72%) with at least one GO term in the biological process category, 194 (57.40%) in the cellular component category and 213 (63.02%) in the molecular function category, while 135 (39.94%) were assigned to GO terms in all three categories. As shown in Figure 6, DEGs in the biological process category were mainly involved in “cellular process” (135 DEGs; 63.68%) and “metabolic process” (134 DEGs; 63.21%), 117 (60.31%), and 164 (84.54%) cellular component DEGs were involved in “intracellular” and “cell part,” respectively. Finally, the most highly represented terms in the molecular function category were “catalytic activity” and “binding,” containing 124 (58.22%) and 128 (60.09%) DEGs, respectively. In addition, GO term enrichment analysis identified a total of eight terms with the correct-*P* = 0.05. Among these, “extracellular region” (25 DEGs; 12.89%), “cell wall” (19 DEGs; 9.79%), “external encapsulating structure” (27 DEGs; 13.92%), and “cell periphery” (29 DEGs; 14.95%) were involved in the cellular component category, and “single-organism biosynthetic process” (24 DEGs; 11.32%), “secondary metabolic process” (19 DEGs; 8.96%), “cell wall organization or biogenesis” (15 DEGs; 7.08%) and “secondary metabolite biosynthetic process” (14 DEGs; 6.60%) were in the biological process category.

The chloroplasts of *drm* were degraded compared to those of “FT.” It is worth noting that GO analysis revealed a number of GO terms related to chloroplast development, including chloroplast thylakoid (nine DEGs), thylakoid (10 DEGs), chloroplast (nine DEGs), and plastid (34 DEGs). In addition, DEGs were assigned to numerous GO terms involved in stress tolerance, such as response to stress (59 DEGs), response to biotic stimuli (20 DEGs), response to fungi (four DEGs), response to hormone stimuli (31 DEGs), response to wounding (two DEGs), response to abiotic stimuli (35 DEGs), response to drugs (three DEGs), response to light stimuli (11 DEGs), response to bacterium (two DEGs), and response to temperature stimuli (two DEGs).

To identify genes involved in important pathways, a total of 183 DEGs were mapped to 150 KEGG pathways. Metabolic pathways, containing 49 DEGs (26.78%), was the most highly represented KEGG pathway, followed by biosynthesis of secondary metabolites (34 DEGs, 18.58%), plant hormone signal transduction (33 DEGs, 18.03%), plant–pathogen interaction (19 DEGs, 10.38%), and microbial metabolism in diverse environments (14 DEGs, 7.65%). Significantly-enriched KEGG pathways are shown in Table 5. In addition, a number of KEGG pathways associated with photosynthetic pigments and photosynthesis were found, including those corresponding to porphyrin and chlorophyll metabolism (one DEG), photosynthesis (two DEGs), and carotenoid biosynthesis (three DEGs); these pathways may be related to the mutant phenotype in *drm*. The results of comparative transcriptome analysis between “FT” and *drm* lay a foundation for further elucidation of gene functions and metabolic pathways in Chinese cabbage.

**Table 5:** **Significantly-enriched KEGG pathways of DEGs in *drm* vs. “FT”**.

**Pathway**	**Pathway ID**	**DEGs**	**Total DEGs**	***P*-values**
Plant hormone signal transduction	ko04075	33	183	8.11E-06
Aminobenzoate degradation	ko00627	10	183	0.000277951
Bisphenol degradation	ko00363	9	183	0.000554168
Limonene and pinene degradation	ko00903	9	183	0.000584729
Polycyclic aromatic hydrocarbon degradation	ko00624	9	183	0.00060052
Glucosinolate biosynthesis	ko00966	5	183	0.000707673
Stilbenoid, diarylheptanoid, and gingerol biosynthesis	ko00945	10	183	0.0013657
Tryptophan metabolism	ko00380	7	183	0.003199293
Linoleic acid metabolism	ko00591	3	183	0.00618751
Biosynthesis of secondary metabolites	ko01110	34	183	0.006752548
Pentose and glucuronate interconversions	ko00040	8	183	0.009201608
alpha-Linolenic acid metabolism	ko00592	5	183	0.01480187
Cyanoamino acid metabolism	ko00460	5	183	0.02163699
Tropane, piperidine, and pyridine alkaloid biosynthesis	ko00960	3	183	0.0316206
Novobiocin biosynthesis	ko00401	2	183	0.03518339
Ascorbate and aldarate metabolism	ko00053	4	183	0.03967568
Starch and sucrose metabolism	ko00500	10	183	0.0442584

*DEGs, the number of DEGs with a specific pathway annotation; Total DEGs: the total number of DEGs with pathway annotations*.

### Analysis of the gene expression patterns by qRT-PCR

**Figure 7:**
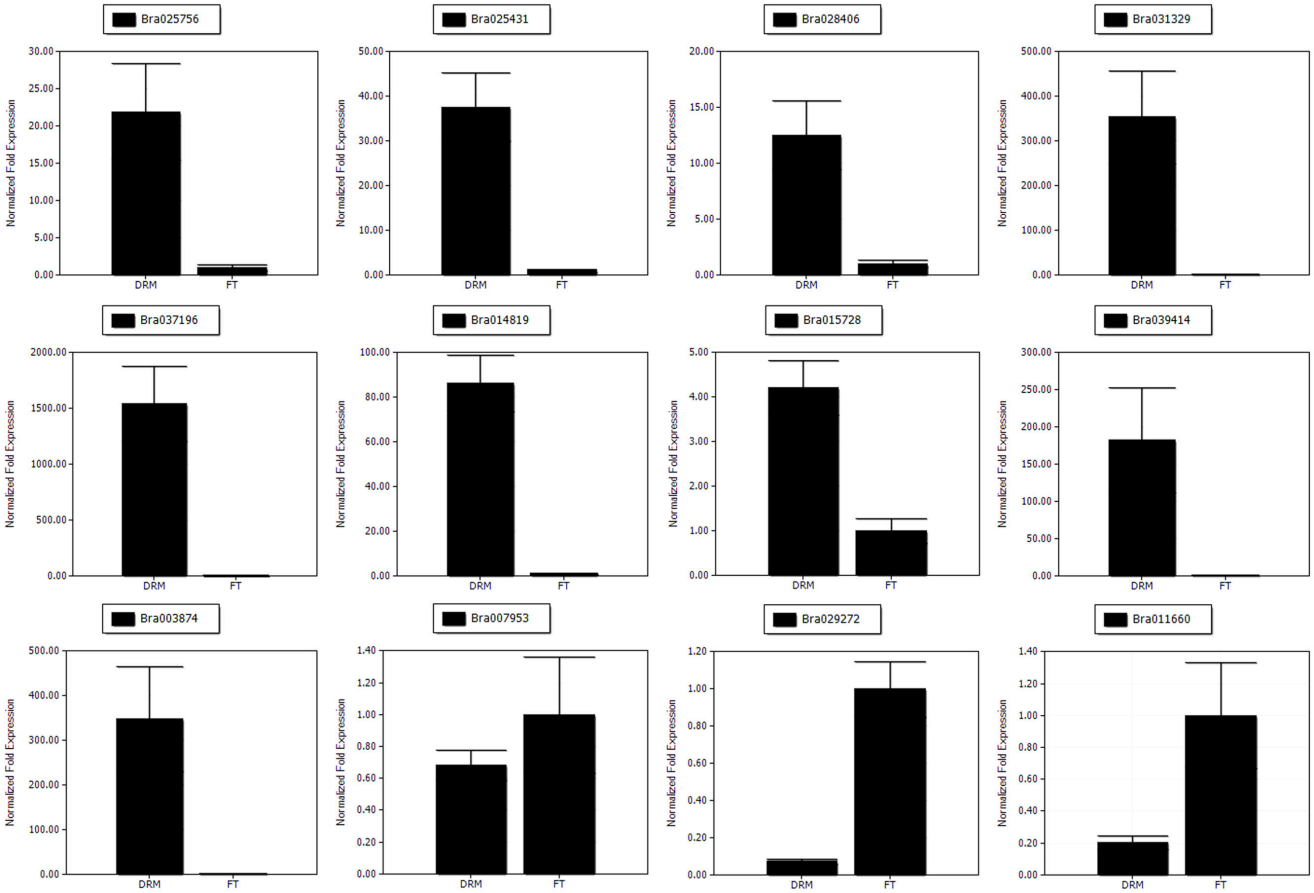
**The qRT-PCR analysis of gene expression patterns**. The relative expression levels for 12 DEGs are shown.

The gene expression patterns determined by qRT-PCR (Figure 7) showed the trends similar to those found in the RNA-Seq data, suggesting that our transcriptome analysis was reliable.

## Discussion

In this study, we characterized the *drm* exhibiting a slow-growing, chlorophyll-deficient phenotype, which was screened by Huang et al. (2014) and derived from the Chinese cabbage DH line “FT” by a combination of isolated microspore culture and radiation treatment (^60^Co γ-rays). Compared with the wild-type “FT” line, the chlorophyll contents were significantly reduced in *drm*, and the chloroplast structure was abnormal. In addition, the chlorophyll fluorescence parameters and the photosynthetic efficiency were significantly reduced in *drm*. These changes in physiological characteristics may inhibit the growth and development of a plant and cause a significant reduction in the important agronomic traits of *drm*, such as leaf length, leaf width of the third true leaves, and plant width at the seedling stage, as well as head weight, head length, and head width at the heading stage. These results suggest that the slow growth and development of *drm* resulted from a significant reduction in these physiological characteristics.

Investigating the differential expression patterns of genes between “FT” and *drm* has important fundamental and practical applications and our data will be valuable for further investigating the growth and development of Chinese cabbage at the molecular level. According to KEGG pathway analysis, one DEG (*Bra025756*) was involved in the porphyrin and chlorophyll metabolism pathway. *Bra025756* encodes chlorophyllase, which plays a critical role in chlorophyll degeneration and is crucial for plant development (Hornero-Méndez and Mínguez-Mosquera, 2002; Okazawa et al., 2006; Yi et al., 2006). Chlorophyllase catalyzes the reaction from chlorophyll to chlorophyllide (Fang et al., 1998; Hörtensteiner, 1999; Tsuchiya et al., 1999). Therefore, the upregulation of the chlorophyllase gene (*Bra025756*) in the *drm* vs. “FT” comparison may accelerate chlorophyll degradation in *drm*, thereby reducing chlorophyll levels and affecting photosynthetic efficiency, ultimately causing the slow growth and development of *drm*. In addition, two other DEGs (*Bra025812* and *Bra026854*) were involved in photosynthesis pathways. However, their functions are currently unknown. Further analysis of these two genes may uncover possible roles in regulating photosynthesis in Chinese cabbage.

SNPs developed based on transcriptomes have been widely employed as powerful molecular markers for genetics, evolution and breeding programs in plants (Blanca et al., 2012). The SNPs in this study were in accordance with those of previous studies, which revealed high frequencies of A/G and C/T transitions in other plant species (Chagné et al., 2008; Blanca et al., 2012). In addition, the SNPs contrasting “FT” and *drm* provide a valuable resource for future molecular marker development and genetic linkage mapping studies in Chinese cabbage, especially some specifically detected SNPs in *drm* may be directly related to the mutant phenotype.

Mutants represent a valuable resource for functional genomics studies and can be used as ideal materials to study a variety of physiological processes (Fambrini et al., 2004; Fujino et al., 2008). Therefore, Chinese cabbage mutants are not only valuable for breeding, but they are also highly useful for identifying the biological functions of selected genes. Leaves are the main sites of photosynthesis and mutations of leaves may significantly influence photosynthesis and even the growth and development of a plant. Consequently, leaf-color mutants represent ideal materials for studying chloroplast development and photosynthesis mechanisms, as well as the functions of genes related to chlorophyll biosynthesis and regulation in plants.

The mutant used in this study exhibits a chlorophyll-deficient phenotype; however, it differs from other chlorophyll-deficient mutants that have been reported (Ansari et al., 2013; Zhu et al., 2014). The mutant in this study not only affects chlorophyll biosynthesis and chloroplast development, but also influences plant growth and development, eventually leading to the production of very small leafy heads. The mutant in this study is closely related to the yield and quality of Chinese cabbage, therefore, further studies of *drm* have important theoretical and practical value. In this study, genetic analysis indicates that the phenotype of *drm* is controlled by a single recessive gene. Fine-mapping of *drm* genes remains a point for further study; gene mapping, and our transcriptome analysis can be combined to predict candidate genes for *drm*, especially the DEGs identified between “FT” and *drm* and the SNPs specifically detected in *drm* based on the transcriptome data. Further investigation of the *drm* gene has significant implications for revealing the molecular mechanism involved in the slow-growing phenotype of *drm* and can also be used in breeding research for Chinese cabbage.

In conclusion, our physiological characterization and comparative transcriptome analysis of “FT” and *drm* have provided important information for better understand the molecular regulatory mechanisms of the phenotypic differences between these lines. Further investigation of the functions of DEGs involved in chlorophyll degradation and photosynthesis, as well as the regulation of chlorophyll metabolic processes, could substantially increase our understanding of growth and development in Chinese cabbage. The resources developed in this study provide a basis for further studies of gene expression and functional genomics in Chinese cabbage.

## Database linking

The transcriptome sequencing data were deposited in the NCBI Gene Expression Omnibus (GEO) Database with the accession number GSE75464.

## Author contributions

HF and ZL conceived and designed the research. SH, DL, and RY performed the research. SH, LH, and XL analyzed the data. SH wrote the manuscript. HF revised the manuscript. All authors discussed the results and approved the final manuscript.

### Conflict of interest statement

The authors declare that the research was conducted in the absence of any commercial or financial relationships that could be construed as a potential conflict of interest.

## Supplementary material

Table S1 **List of all genes detected in the “FT” and *drm* libraries (XLS)**.

Table S2 **Summary of extended genes detected in the “FT” (FT2, FT4, FT5) libraries (XLS)**.

Table S3 **Summary of extended genes detected in the *drm* (DRM2, DRM4, DRM5) libraries (XLS)**.

Table S4 **List of novel transcripts detected in the “FT” and *drm* libraries (XLS)**. Note: The meaning of numbers in the columns labeled “Blocks” were the number of exons in the novel transcript; “Size” were the sizes of each exon in the novel transcript (comma-separated); and “Starts” were the starting positions of each exon on the chromosome (comma-separated).

Table S5 **Summary of SNPs detected in the “FT” and *drm* libraries (XLS)**.

Table S6 **Gene IDs and expression data for DEGs in the *drm* vs. “FT” comparison (XLS)**.

Table S7 **Primers used for qRT-PCR analysis (XLS)**.
